# Ratiometric Fluorescent pH Sensing with Carbon Dots: Fluorescence Mapping across pH Levels for Potential Underwater Applications

**DOI:** 10.3390/nano14171434

**Published:** 2024-09-02

**Authors:** Wiktoria Karolina Szapoczka, Chiara Olla, Cristina Carucci, Adam Leo Truskewycz, Tore Skodvin, Andrea Salis, Carlo Maria Carbonaro, Bodil Holst, Peter James Thomas

**Affiliations:** 1 Department of Physics and Technology, University of Bergen, 5007 Bergen, Norway; bodil.holst@uib.no; 2 Department of Physics, University of Cagliari, Cittadella Universitaria, I-09042 Monserrato, Italy; cm.carbonaro@dsf.unica.it; 3 Department of Chemical and Geological Sciences, University of Cagliari, Cittadella Universitaria, I-09042 Monserrato, Italy; cristina.carucci@unica.it (C.C.); asalis@unica.it (A.S.); 4 Department of Biomedicine, University of Bergen, 5009 Bergen, Norway; adam.truskewycz@uib.no; 5 Department of Chemistry, University of Bergen, 5007 Bergen, Norway; tore.skodvin@uib.no; 6 NORCE Norwegian Research Centre AS, 5008 Bergen, Norway; peth@norceresearch.no

**Keywords:** carbon dots, fluorescence mapping, pH sensing, ratiometric fluorescence, seawater acidification

## Abstract

Ocean acidification has become a major climate change concern requiring continuous observation. Additionally, in the industry, pH surveillance is of great importance. Consequently, there is a pressing demand to develop robust and inexpensive pH sensors. Ratiometric fluorescence pH sensing stands out as a promising concept. The application of carbon dots in fluorescent sensing presents a compelling avenue for the advancement of pH-sensing solutions. This potential is underpinned by the affordability of carbon dots, their straightforward manufacturing process, low toxicity, and minimal susceptibility to photobleaching. Thus, investigating novel carbon dots is essential to identify optimal pH-sensitive candidates. In this study, five carbon dots were synthesized through a simple solvothermal treatment, and their fluorescence was examined as a function of pH within the range of 5–9, across an excitation range of 200–550 nm and an emission range of 250–750 nm. The resulting optical features showed that all five carbon dots exhibited pH sensitivity in both the UV and visible regions. One type of carbon dot, synthesized from m-phenylenediamine, displayed ratiometric properties at four excitation wavelengths, with the best results observed when excited in the visible spectrum at 475 nm. Indeed, these carbon dots exhibited good linearity over pH values of 6–9 in aqueous Carmody buffer solution by calculating the ratio of the green emission band at 525 nm to the orange one at 630 nm ($I_{525\;\mathrm{nm}}$/$I_{630\;\mathrm{nm}}$), demonstrating highly suitable properties for ratiometric sensing.

## 1. Introduction

Ocean acidification is a pressing global issue, primarily driven by increased carbon dioxide emissions from human activities [1,2,3,4]. As CO_2_ accumulates in the atmosphere, a significant portion is absorbed by the oceans, leading to chemical reactions that reduce seawater pH [1,2,5]. This process threatens marine ecosystems, particularly calcifying organisms such as corals and shellfish, which play crucial roles in marine food webs and biodiversity [6,7,8,9]. The broader impacts of ocean acidification extend to the socio-economic structure of communities reliant on marine resources, highlighting the urgent need for effective monitoring and mitigation strategies [8,9,10]. In the pursuit of understanding and addressing the challenges posed by ocean acidification, the scientific community and industry stakeholders have shown increasing interest in developing advanced technologies for accurate and reliable pH monitoring.

Historically, the most widely used pH sensor across various industries has been the classical pH electrode [11,12,13]. Although inexpensive, it poses several disadvantages, such as the tendency to drift, requiring expert care for recalibration [11] and frequent electrolyte replenishment [12]. Additionally, pH electrodes are less effective in high-salinity environments due to unstable junction potentials at the reference electrode [14,15,16], making them more suitable for spot sampling than continuous monitoring.

**Figure 1:**
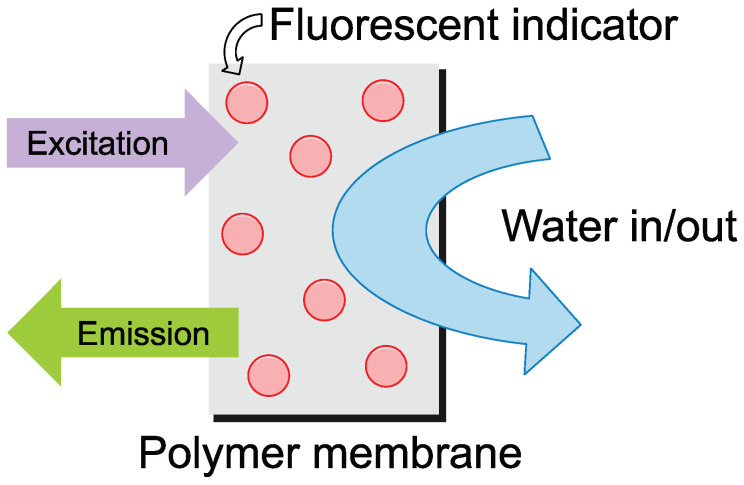
General idea for an optical pH sensor. A pH-sensitive fluorescent indicator is embedded in a polymer membrane, and as the water comes in contact with the film, it is excited. From the measured emission, it is possible to assess the pH of the water in which the film is submerged.

To overcome these limitations, several sensing technologies have been developed, including spectrophotometric sensors and ion-sensitive field effect transistors. These technologies, while accurate, require periodic refilling of agents and a reference electrode, respectively [17,18,19,20,21]. Optical sensors have emerged as a viable alternative due to their cost-effectiveness, low power consumption, and long-term stability [22,23,24,25]. They hold significant value in several fields, notably biomedicine [23,24,26] and environmental research [12,27,28]. These sensors typically consist of a sensing film containing a pH-sensitive indicator embedded within a medium that allows for ion penetration (Figure 1). As the pH of the surrounding liquid shifts, it alters the specific optical characteristics of the indicator, which are then accurately measured using optoelectronic techniques. Fluorescent pH sensors offer remarkable sensitivity and selectivity, along with superior spatial and temporal resolution, enabling real-time in situ imaging. In the field of oceanography, fluorescence-based optical oxygen sensors are already widely recognized and utilized [12,28,29].

Among the various emerging technologies, ratiometric pH sensing is advantageous due to its potential for high precision and stability in environmental measurements [30,31,32,33]. A ratiometric pH sensor incorporates a pH-sensitive indicator that emits light at two or more distinct wavelengths under specific excitation light. As the pH changes, the intensities of these emissions vary in a predictable and opposite way. By calculating the ratio of these intensities, the pH of the environment can be determined more precisely.

The key feature of ratiometric pH sensing is that it compares the intensity ratios of fluorescence emissions rather than relying on the absolute intensity at a single wavelength, which can be influenced by various factors like light source variations and environmental conditions [34,35]. A ratiometric pH sensor is less prone to being affected by the concentration of the sensing nanoparticle since it measures the ratio of two fluorescence intensities at two different wavelengths and compares them [34,36]. Nevertheless, very high or very low concentrations of the sensing nanoparticle can affect the performance, so an optimal concentration should be used when designing a ratiometric pH sensor to ensure that the sensor is in its dynamic range, where the pH-induced changes in the fluorescence ratio are most accurate and linear [37,38]. To secure the sensor’s longevity, it can be beneficial to design a solution that ensures a stable concentration of CD at all times. One potential solution could be to embed the nanoparticles in a film, preventing the material from leaching into the solution. Currently, there is significant work towards developing innovative optical pH sensors, underscoring the need for new sensing materials [39,40].

Carbon dots (CDs) have shown considerable potential for ratiometric pH sensing [33,41,42]. CDs are a novel class of nanomaterials, characterized by impressive photoluminescent properties [43]. Since their accidental discovery in 2004, CDs have attracted substantial interest in various fields, including bioimaging, drug delivery, and environmental monitoring [44]. Their luminescent efficiency, chemical stability, low toxicity, ease of production, and unique optical properties [42,45,46] make them ideal potential indicators for long-term monitoring applications [46,47,48].

Several CD-based ratiometric pH sensors have been reported, aiming for use in intracellular pH monitoring, bioimaging, detection of cancerous cells, and sensing of L-lysine, among other applications [33,41,42,49,50,51,52]. The ability of CDs to track pH changes in real-time in biological systems further highlights their potential for underwater pH monitoring [49]. Despite their promising attributes, the application of CDs in underwater pH sensing remains largely unexplored and underutilized.

In our previous work, we reported the characterization of fluorescence intensity (FI) and lifetime (FL) response of four novel solvothermally synthesized CDs [47]. A fifth CD was used as a reference, synthesized following a published method [26]. The characterization aimed to determine if any of the CDs were suitable candidates for an FL-based optical sensor, but it focused only on a single excitation wavelength (452 nm) and explored a limited range of emission and decay times. None of the CDs showed strong enough FL dependency within the investigated optical range to move forward with developing a reliable FL-based pH sensor. The work also included an investigation of how environmental factors such as a low temperature (3 °C), buffer salinity (3.5%—the salinity of seawater), and photobleaching affect the resilience of CDs. We were able to show that after being exposed to the excitation source for at least 52 h, the CDs showed little to no change in their optical response, showcasing the suitability of these nanoparticles for use in continuous pH monitoring. The overall fluorescence response of the CDs is positively affected by low temperatures and salinities of the buffer, generally increasing the fluorescence intensity, which is advantageous for use in marine applications. Again, using a ratiometric pH sensor, one measures the ratio between two intensities, so any overall change in the response would not affect the measurement. Additionally, the stability of CDs has been reported in several other papers, including the work performed using a CDs-based ratiometric pH sensor [33,45,48,53]. Our study concluded that further research on the optical properties of CDs is needed to fully investigate these CDs’ suitability as fluorescent sensors.

The intensity of fluorescence is affected by the concentration of CD, as mentioned above, but the pH sensitivity trend across a pH range will not change unless the concentration becomes extremely low. Fluorescence lifetime is not affected by the concentration. For a potential ratiometric pH sensor, it would be beneficial to design a solution that would ensure a stable concentration of CD at all times to secure the longevity of the sensor without a constant need for recalibration.

**Figure 2:**
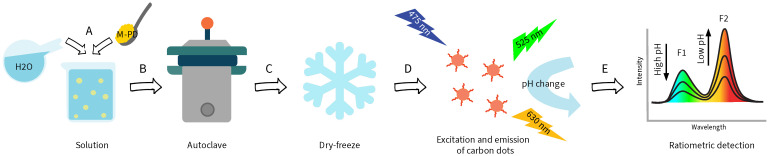
Synthesis of carbon dot CD04: (**A**) m-Phenylenediamine (m-PD) is dissolved in distilled water, (**B**) the solution is transferred to an autoclave and heated at high temperature and pressure, (**C**) the solution undergoes dry-freezing, (**D**) fluorescent CDs are formed and excited at different wavelengths (here: $\lambda_{\mathrm{exc}}$ = 475 nm), and the resulting emission is recorded at different pH values, (**E**) detection and analysis of the emission by calculating the ratio between the two intensity peaks, F1 and F2.

In this paper, we present a novel investigation of the optical response of five CDs, performed using fluorescence mapping (Figure 2). The five CDs were synthesized using a straightforward solvothermal method, as described in the previous work. Their fluorescence responses were investigated by exciting the CDs in the 200–550 nm range and analyzing the corresponding emission bands in the 250–750 nm range from pH 5 or 6 to pH 9 in Carmody buffer solution. Carmody buffer solution was chosen for its ease of production and wide pH range. The pH range was selected to encompass values relevant for possible future sensing applications in marine waters, where the pH is approximately 8.2 [54,55]. Furthermore, pH values in freshwater range from 6.5 to 8.5 [56,57,58]. By expanding the excitation range, we were able to observe that all the synthesized CDs exhibited pH sensitivity, and four of these CDs showed emission peaks in both the visible and UV spectra. Notably, CD04, synthesized from m-phenylenediamine (m-PD), was found to have promising ratiometric pH sensing properties, demonstrating pH sensitivity at four different excitation wavelengths. Following this, the fluorescence response of CD04 was further mapped at additional pH values of 7 and 8. The intensity ratios at $\lambda_{\mathrm{exc}}$ = 475 nm ($I_{525\;\mathrm{nm}}$/$I_{630\;\mathrm{nm}}$) exhibited good linearity between pH 6 and 9, indicating promising ratiometric features.

## 2. Materials and Methods

### 2.1. Materials

Ammonium fluoride, boric acid, citric acid (monohydrate) (CA), disperse blue 1 dye (DB1), N,N-Dimethylformamide (DMF), ethanol, m-Phenylenediamine (m-PD), phloroglucinol (PG), sulfuric acid, tertiary sodium phosphate, and toluene were purchased from Sigma Aldrich (St. Louis, MO, USA). Ultrapure water (Milli-Q) was utilized in the synthesis.

### 2.2. Synthesis of CDs

The synthesis methods for the five carbon dots (CDs) used in this study follow the procedures reported in our previous work [47]. Briefly:

1. CD01: A solution was prepared by dissolving 0.3 g of disperse blue 1 dye (DB1) and 0.7 g of phloroglucinol (PG) in 40 mL of ethanol containing 7.5% sulfuric acid. This solution was transferred to a Teflon-lined hydrothermal vessel and heated to 185 °C for 3.5 h. After cooling to room temperature, the solution was resuspended in a mixture of 100 mL of toluene and water in a 1:1 volume ratio. The toluene fraction was removed, and the water-soluble fraction was dried under a fume hood, resuspended in 10 mL of ethanol, diluted to 100 mL with ultrapure water, filtered through a 0.2 µm syringe filter, dialyzed (MWCO 3500) against ultrapure water for 72 h, and freeze-dried.
2. CD02: Following the removal of the toluene fraction, the solvent was evaporated under a fume hood. The resulting fraction was dissolved in ethanol, dialyzed (MWCO 3500) using ultrapure water for 72 h, and freeze-dried.
3. CD03: A solution comprising 0.3 g of DB1, 0.4 g of citric acid, and 0.1 g of ammonium fluoride in 30 mL of DMF was prepared. This solution (ammonium fluoride not dissolved) was added to a Teflon-lined hydrothermal vessel and heated to 180 °C for 3 h. The resulting solution was dried under a fume hood on a plastic plate, resuspended in water, dialyzed (MWCO 3500) against ultrapure water for 72 h, and freeze-dried.
4. CD04: A total of 1 g of *m*-phenylenediamine (*m*-PD) was dissolved in 40 mL of water. The solution was transferred into a Teflon-lined hydrothermal vessel and heated at 180 °C for 3 h. After cooling to room temperature, the resulting solution was dialyzed (MWCO 3500) using ultrapure water for 72 h and freeze-dried. This procedure was adapted from the literature [26].
5. CD05: A total of 1 g of *m*-PD and 1 g of PG were dissolved in 40 mL of DMF containing 0.6% sulfuric acid. The solution was added to a Teflon-lined hydrothermal vessel and heated to 185 °C for 3.5 h. The resulting solution was dialyzed (MWCO 3500) against ultrapure water for 72 hours and freeze-dried.

### 2.3. Preparation of Samples

Ethanol was added to all five CDs to achieve a 1 mg/mL concentration. For the measurements, the concentration of the CDs in Carmody buffer solutions varied between 0.6 mg/L and 20 mg/L. At the highest concentration, each sample consisted of 80 µL of the ethanol solution mixed with 4 mL of Carmody buffer. The concentrations were carefully selected to avoid oversaturating the instrument while ensuring a sufficiently strong signal. This was necessary because the fluorescence intensity is directly proportional to the concentration of CD, and an excessively high concentration can oversaturate the instrument. The pH sensitivity trend across a pH range does not change with concentration unless the concentration is extremely high or low. If the concentration is too high, fluorescence quenching or reabsorption can occur, resulting in intensities that are lower than expected.

### 2.4. Optical Characterization of CDs

UV–Vis-NIR absorbance spectra were collected (applying baseline corrections) using a Jasco V-750 spectrophotometer with a spectral bandwidth of 2 nm in the 200–800 nm range.

Three-dimensional fluorescence mapping of samples was performed using a spectrofluorometer, a Jasco FP-8050, with a 450 W Xenon lamp as the excitation source. The maps were collected using an excitation range of 200–550 nm and an emission range of 250–750 nm with a 2.5 nm spectral bandwidth for excitation and emission.

Time-resolved photoluminescence measurements were performed by exciting the samples with 200 fs long pulses delivered by an optical parametric amplifier (Light Conversion TOPAS-C) pumped by a regenerative Ti:Sapphire amplifier (Coherent Libra-HE). The repetition frequency was 1 kHz, and the PL signal was recovered by a streak camera (Hamamatsu C10910) equipped with a grating spectrometer (Princeton Instruments Acton SpectraPro SP-2300). All the TR-PL measurements were gathered by exciting the samples in the front-face mode to avoid the inner filter effect. The solutions were placed in a 10 mm quartz cuvette. Proper optical filters were applied when needed.

### 2.5. Zeta Potential of CDs

A volume of 4 mL of CD solution was transferred to a 10 mL beaker and acidified to pH 1–2 by adding a small volume of 0.1 M HCl. A dry syringe was used to rinse and fill a DTS1070 disposable cell for the measurement of zeta potential (ζ). After the measurement, the CD solution was carefully recovered in the titration beaker. The CD solution was then titrated with 0.1 M NaOH. After each NaOH addition, the pH and the corresponding zeta potential were measured up to pH 10–11. Zeta potential measurements were carried out using a Malvern Zetasizer Nano ZSP equipped with a laser (λ = 633 nm). A pH meter Metrohm equipped with a microelectrode code 6.0234.100 calibrated with buffers at pH 4, 7, and 10 was used for the pH measurements. At least three zeta potential measurements were made at each pH value, and the average value and the error bar (standard deviation) were reported.

## 3. Results and Discussion

### 3.1. Optical Characterization of CDs in the Buffer Solution

In our previous study [47], we explored the optical properties of five types of solvothermally synthesized carbon dots (CD01–CD05) when excited with monochromatic light at 452 nm, with emissions observed above 500 nm. The CDs were structurally analyzed using Fourier transform infrared (FTIR) spectroscopy, which identified a complex surface chemistry including hydroxyl (-OH), amine (-NH), and carboxyl (C=O) groups that underscored the CDs’ suitability for a wide range of applications.

**Figure 3:**
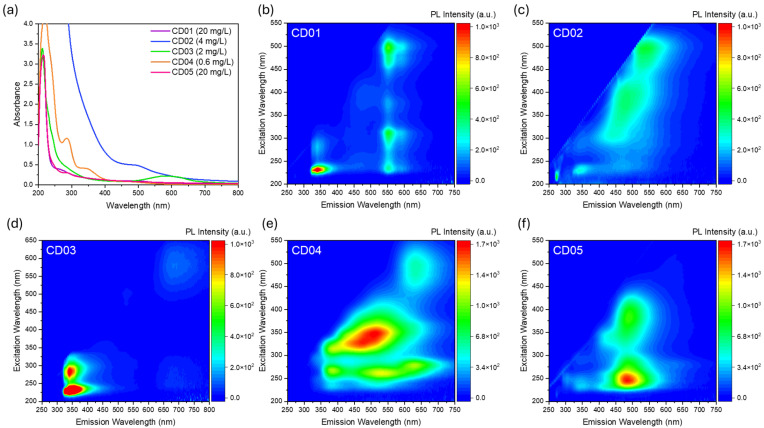
Optical absorption spectra (**a**) and excitation and emission maps (**b**–**f**) of the series of carbon dots at pH 9.

In the current investigation, we extended our analysis to cover the entire visible spectrum to determine the optimal excitation wavelength. Initially, we analyzed CDs dispersed in 100 mM Carmody buffer. Our analysis began with solutions at a basic pH of 9, where the CDs typically exhibited the highest photoluminescence (PL) intensity within the studied pH range [47]. The concentrations of CDs varied from 0.6 mg/L (CD04) to 20 mg/L (CD01 and CD05) to prevent instrument saturation, taking into account the differing absorption intensities of the CDs. Figure 3 illustrates the distinct absorption and excitation/emission characteristics of the CD series across the UV–visible range.

The optical properties of this series of CDs were clearly influenced by their different precursors and synthetic procedures.

CD01 is derived from the water-soluble fraction of the chemical reaction between phloroglucinol (PG), a small symmetrical molecule, and disperse blue 1 dye (DB1), an amino-substituted anthraquinone. It exhibits a primary absorption peak in the UV region at 215 nm, with less-intense shoulders at 270 nm and 500 nm (Figure 3a, purple line). This sample shows one narrow emission channel in the near-UV (340 nm, excited at 230 nm) and two in the green–yellow region (550 nm and 585 nm, excited at 230 nm, 300 nm, and 500 nm), as depicted in Figure 3b and Figures S1a and S2a. The optical features of CD01 resemble those of both its precursors. Indeed, although little information is available regarding the optical emission of DB1, which is not a common CD precursor compared to other dyes [59,60,61], the absorption properties of the anthraquinone family are well-documented. These compounds are known for their colorful dyes, easily tuned according to specific substituents [62,63]. For DB1, the UV absorption channels are attributed to $\pi -\pi^{*}$ transitions, while the visible ones at around 600–630 nm, responsible for the blue color of the dye, are due to intramolecular charge-transfer transitions associated with charge migration from the amino group to the carbonyl groups [64]. In CD01, no distinguishable absorption in the 600–630 nm region was recorded. However, the UV–Vis absorption is compatible with the formation of the aromatic system originating from this precursor and could be linked to the intense UV emission observed, which is also present in DB1-derived CD03 (*vide infra*).

PG is a well-known CD precursor that is capable of producing narrow-bandwidth emissive CDs that are suitable for lasing applications, depending on the size and shape of the aromatic domain [65,66,67]. CD01 shows two narrow emission bands in the green–yellow region, with multiple excitation channels, which are similar to those reported in the literature using similar synthetic procedures. Previous structural measurements [47] revealed a bimodal size distribution of the CD01 nanoparticles, indicating the presence of two predominant sizes. This could be due to the different reactivity of the precursors, leading to various reaction pathways and different emitting species [68,69,70,71]. Some species are likely associated with DB1-derived structures emitting in the UV region, as suggested bythe comparison of CD01 and CD03, while others could be responsible for the typical emission of PG-based CDs in the visible region.

The toluene-soluble fraction from the same synthesis, named CD02, despite its high absorption intensity extending into the UV region and a broad shoulder at around 500 nm (Figure 3a, blue line), shows less-efficient luminescence compared to CD1 (Figure 3c and Figures S1b and S2b), also considering that it has a a four times lower concentration by weight. Its three main emissions are displayed at 340 nm (excited at 300 nm), 485 nm (excited at 400 nm), and 540 nm (excited at 500 nm), differing from CD01 despite the same synthetic precursors, reflecting variable emission channels that were separated post-synthesis. Indeed, in the previous article, we measured a bimodal size distribution for the CD01, with peaks at about 1.5 nm and 5.5 nm, while CD02 exhibited a monomodal distribution, peaking at 1.6 nm [47]. According to the optical features analyzed in this work, we hypothesize that the smaller particles are mainly related to the PG precursor, leading to visible emission centers. On the other hand, DB1-derived structures are larger in size and could be responsible for the emissions in the UV range.

CDs synthesized from citric acid (CA) and DB1, referred to as CD03, exhibit distinct absorption peaks at 210 nm, a shoulder at 290 nm, and a minor contribution at 585 nm (Figure 3a, green line), corresponding to their emission features (Figure 3d and Figures S1c and S2c). Specifically, CD03 shows two primary emissions: a strong UV emission at 340 nm (excited at 230 or 280 nm) and a secondary, less-intense, red emission at 685 nm (excited at 580 nm). The UV emission characteristics strongly resemble those of CD01, likely due to the shared reagent DB1 used in their synthesis. The DB1’s influence on the absorption properties is evident based on the presence of the band in the orange range, probably linked to the faint red emission.

CD03 does not display the typical optical features of CA-derived CDs, which generally emit in the blue range [72,73,74]. We can exclude the presence of molecular residues from the DB1 precursors due to dialysis and the TEM analysis performed in our previous work (Figure 2, [47]) to assess the particle size and morphology, which confirms the formation of carbon nanostructures. Even in this case, we previously observed the presence of two size distributions peaking around 1.5 nm and 5 nm, suggesting different reaction pathways during synthesis [47]. The preservation of DB1 properties may be due to its low reactivity, with CA cyclization forming the core structure that incorporates DB1. Attempting to combine the indications gathered from these parent three samples, we might hypothesize that the smaller nanoparticles are related to the carbonization of the smaller organic molecules such as PG and CA, whilst the larger ones are obtained via anchoring or embedding the larger DB1 dye in the carbon network in a core–shell-like model, as hypothesized by several theoretical studies [75,76,77,78]. Under this assumption, the UV excitation–emission features observed in CD01 and CD03 are provided by the DB1 in the larger nanoparticles; the visible properties of CD01 and CD02, on the contrary, pertain to the smaller nanoparticles.

The simplest solvothermal synthesis among the five CDs, involving m-phenylenediamine (m-PD) and ultrapure water, as reported in the literature [26], produced CD04, which is the most optically diverse sample. CD04 has absorption peaks in the UV spectrum at 220 nm, 285 nm, and 340 nm, with a faint shoulder at about 450 nm (Figure 3a, orange line). This sample presents multiple broad excitation–emission channels across the visible spectrum (Figure 3e and Figures S1d and S2d): UV emission at 385 nm (excited at 270 and 320 nm), a broad blue–green emission ranging from 420 nm to 550 nm (mainly excited between 320 nm and 365 nm, but with less-intense excitation channels at about 270 nm), and a secondary orange emission at 630 nm (excited at 280 nm, 360 nm and 490 nm).

The isomers of PD, including m-PD, are currently being researched for the synthesis of CDs. This is due to the high reactivity of their amino groups, which allow PD to form various oxidation products via oxidation and polymerization across a wide range of temperatures. These oxidation products can undergo further cross-linking, carbonization, or polymerization to produce CDs [79]. Consequently, for the highly varying nature of PD-based products, CDs are typically employed in sensing and pH response [80,81,82]. Thus, the multiple emitting centers may represent the multiplicity of the way in which m-PD molecules were able to combine across the synthesis, and their coexistence in the mixture makes it possible to obtain multiple centers that can be separated by chromatography, as reported in the literature [83]. This hypothesis is supported by the lack of a Gaussian size distribution that showed nanoparticles in our sample ranging from 1 nm to 6 nm [47].

The last sample, CD05, derived from PG and m-PD, shows a similar optical absorption profile (Figure 3a, pink line) to that of CD01, also derived from PG, with a common main peak at 215 nm, but with an additional shoulder at 290 nm. CD05 (Figure 3f and Figures S1e and S2e) exhibits a principal broad emission in the blue region at 485 nm, with an excitation range from 225 to 425 nm, and a secondary UV emission at 340 nm.

In this case, we can clearly distinguish two broad excitation channels that lead to the same principal emission in the blue–green region but possibly originate from different sources. This may be due to the multiple products obtained from recombining the highly active m-PD and phloroglucinol. Additionally, this sample was found to show large clusters of CDs ranging from 5 to 35 nm [47], which can result in unique optical features.

### 3.2. Analysis of Optical Response as a Function of pH

**Figure 4:**
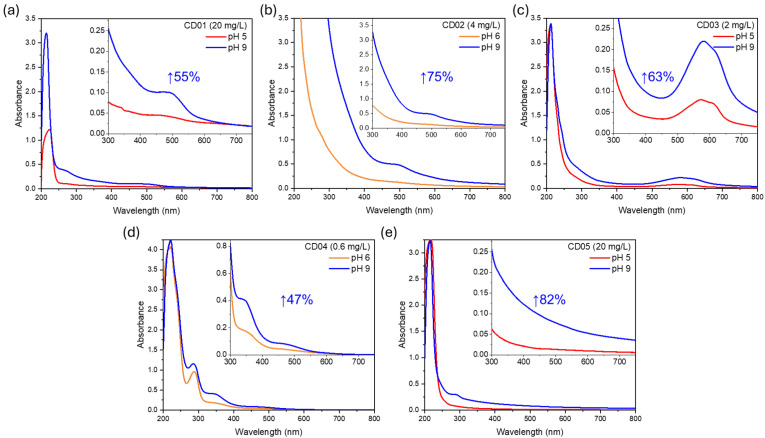
Optical absorption spectra from 200 to 800 nm across pH 5/6 to 9 for (**a**) CD01, (**b**) CD02, (**c**) CD03, (**d**) CD04, and (**e**) CD05. The inset shows a zoom of the absorption spectra in the visible region.

To investigate the pH sensitivity and corresponding optical response of the CDs, we first conducted UV–Vis absorption measurements under different pH conditions (Figure 4).

The CDs were dissolved in Carmody buffer solutions at pH 5 or 6 and compared to those at pH 9. The choice of analyzing pH 5 or 6 was dictated by their emission properties; in some cases, the emissions at pH 5 were too low to be recorded with sufficient intensity. Absorption measurements indicate that all CDs exhibit a general increase in absorbance intensity across the entire investigated range by enhancing the pH from 5–6 to 9 while maintaining their absorption profiles. The extent of absorbance increase in the visible spectrum (inset) varies among the different types of CDs, ranging from a 47% increase in the area in CD04 to an 82% increase in CD05.

**Figure 5:**
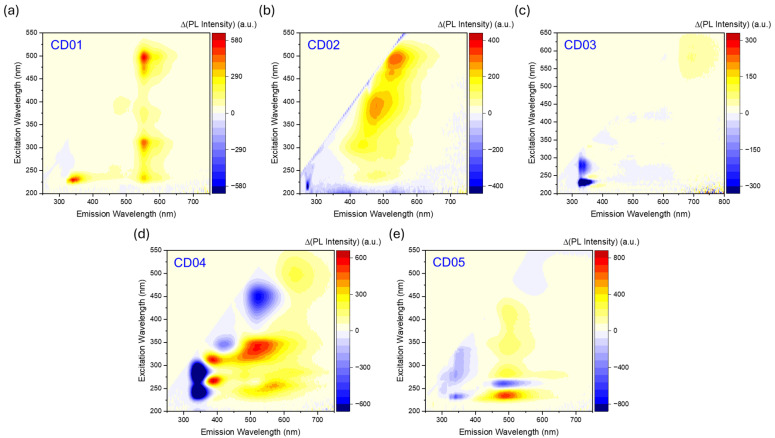
Differential excitation–emission maps highlighting pH-dependent variations in PL intensity of (**a**) CD01, (**b**) CD02, (**c**) CD03, (**d**) CD04, and (**e**) CD05.

The scenario appears clearer when analyzing the differential excitation–emission maps (Figure 5), obtained by subtracting the map at pH 5 or 6 from the one at pH 9 (Figure S3), which shows the intensity variation. These differential maps highlight the centers that increase (shown in red) or decrease (shown in blue) in PL intensity when changing from pH 5 to pH 9, providing insights into the pH sensitivity of the CDs’ optical properties. For a material to be suitable as a ratiometric pH sensor, it must exhibit two or more emission bands under a specific excitation wavelength, with their intensity ratio varying inversely with pH changes.

Unfortunately, the first three CDs in the series do not exhibit these properties at any excitation wavelengths investigated in both the UV and visible ranges (Figure 5a–c). However, they can still be used as suitable fluorescent pH sensors, since their luminescent properties change with pH (Figure S4a–c).

For instance, CD01 exhibits multiple emitting bands at its main excitation wavelengths (230 nm and 500 nm), with all bands increasing in intensity as the pH rises. While the UV bands keep the same shape by decreasing the pH, the green–yellow bands cannot be detected at low pH values (Figure 5a and Figure S4a). Notably, the emission in the visible region, attributed to PG-derived channels, is completely suppressed in the acidic environment at both excitation wavelengths. A similar behavior is observed in CD02, which shows two broad emission contributions, peaking at 440 nm when excited at 300 nm and at 485 nm when excited at 400 nm. Both PL bands retain their shape but decrease in intensity as the pH lowers (Figure 5b and Figure S4b), though they are not entirely suppressed. Consequently, these samples, particularly CD01, when excited in the visible range at 500 nm, can function effectively as a classical fluorescent pH sensor but not as a ratiometric one. CD03 (Figure 5c and Figure S4c) shows a potential ratiometric trend when excited at 230 nm, with decreased UV emission and increased red emission. However, the red emission is too low in intensity and too spectrally distant from the UV emission to be a viable sensing candidate. In addition, the excitation light in the far UV is not appealing nor suitable for practical applications. When excited at 580 nm, the variation in the red band with pH becomes more evident.

One should note that the same UV emission ascribed to DB1 in CD01 and CD03 undergoes opposite variation as a function of the pH change. Since we assigned the optical features to DB1 molecules bonded to different carbon cores in the two samples, we can explain this trend by considering the different functional groups observed using FTIR in the two samples [47]. Indeed, CD03 has a larger content of COOH groups than CD01, thus providing a different sensitivity to the pH environment. A similar explanation also holds for the trend of visible emissions recorded in CD01 and CD02. This emission was ascribed to small PG-related carbon structures with variable contents of auxochromic OH edge groups [47]. As the pH decreases, the deprotonation of these groups decreases, leading to an overall reduction in the emission in the visible range.

**Figure 6:**
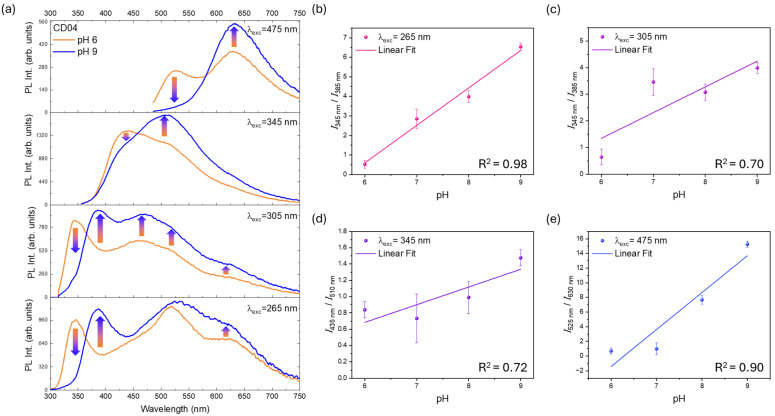
(**a**) PL spectra of CD04 excited at different excitation wavelengths at pH 6 (in orange) and pH 9 (in blue). (**b**–**e**) Plots of the values of the intensity ratio of the main ratiometric peaks from pH 6 to pH 9 with statistical errors from independent experiments.

The most promising sample is CD04 (Figure 5d), which exhibits multiple emitting centers with varying behaviors depending on pH. The sample demonstrates a suitable range of excitation wavelengths for ratiometric analysis, as shown in Figure 6a.

At an excitation wavelength of 265 nm, the UV band at 345 nm nearly disappears when passing from acidic to basic conditions, while bands at 385 nm and 625 nm increase, and the band at 520 nm remains approximately constant in PL intensity. Moreover, a pH calibration curve in the Carmody buffer solution was reported in Figure 6b by calculating the ratio of the PL intensity of the main peaks ($I_{345\;\mathrm{nm}}$/$I_{385\;\mathrm{nm}}$), which exhibits very good linearity across the pH range (data extracted from the excitation and emission maps, Figures S3–S5).

A similar trend is observed with excitation at 305 nm, where the UV bands at 345 nm and 385 nm follow the same trend as the excitation at 265 nm. In addition, a relative increase in the blue–green region (470–520 nm) is observed, as well as for the orange band at 625 nm. However, the linearity of the main peak ratio ($I_{345\;\mathrm{nm}}$/$I_{385\;\mathrm{nm}}$, Figure 6c) is less optimal under this excitation wavelength.

When excited at 345 nm, the pH variation affects the spectral shape of CD04, leading to a decrease of the band at 435 nm and an increase at 510 nm. Although this excitation wavelength could be appealing because some UV LEDs are already commercially available, the linearity of the $I_{435\;\mathrm{nm}}$/$I_{510\;\mathrm{nm}}$ ratio still remains low for a ratiometric pH device (Figure 6d).

The best results are obtained for an excitation wavelength of 475 nm in the visible region. Here, the emission peak at 525 nm in the green region decreases with increasing pH, while the band in the orange range at 630 nm increases. These bands are distinct and can be deconvolved as two emission bands at pH 6 and 9 (Figure S6). The use of visible light enhances the sensor’s suitability, and the linearity of the $I_{525\;\mathrm{nm}}$/$I_{630\;\mathrm{nm}}$ ratio is good (Figure 6e). The ratiometric nature of this CD, particularly at varying pH levels, underlines its potential for developing more sophisticated ratiometric seawater sensors.

CD04 is synthesized from nitrogen-rich m-PD, and several dual-emissive N-doped CDs have been successfully synthesized by using nitrogen-rich precursors [35,84,85,86,87,88]. The FTIR spectrum confirms the presence of C-N bonds [47]. This nitrogen content contributes to the ratiometric behavior of CD04. Changes in the pH environment induce protonation and deprotonation of the nanoparticlew, affecting the fluorescence of their multiple emitting states, which respond differently to varying environmental conditions. Functional groups such as −COOH, −OH, and $-NH_{2}$, previously identified, can create surface states responsible for pH-sensitive emission at longer wavelengths [47,84,89,90]. These functional groups on the shell are easily ionizable and can emit fluorescence independently of the core. As excitation wavelengths increase, only the surface states are excited due to their lower bandgap, while the core remains unexcited due to its larger bandgap [84]. Combined with fluorescence quenching at the nanoparticle shell, this effect contributes to the ratiometric nature observed in some CDs [85].

CD05 (Figure 5e and Figure S4d), also derived from m-PD, shows a suitable ratiometric behavior with decreasing near-UV contributions (300–400 nm) and increasing visible contributions (400–700 nm) with increasing pH values. However, its application as a ratiometric sensor is challenging due to the need for far UV excitation light, which is less convenient compared to visible light. Nonetheless, it is particularly interesting that the same emitting channels can exhibit varying intensities based on pH by altering the excitation wavelength, as observed for the main emission around 500 nm. This allows for the potential development of an unconventional PLE ratiometric sensor with fixed emission and variable excitation, though it is less practical compared to traditional ratiometric sensors.

In order to better understand the variations in the PL features with pH, we performed time-resolved photoluminescence (TR-PL) measurements, exciting the samples at 360, 410, and 500 nm according to the excitation channels of the most interesting emission bands for ratiometric purposes.

**Figure 7:**
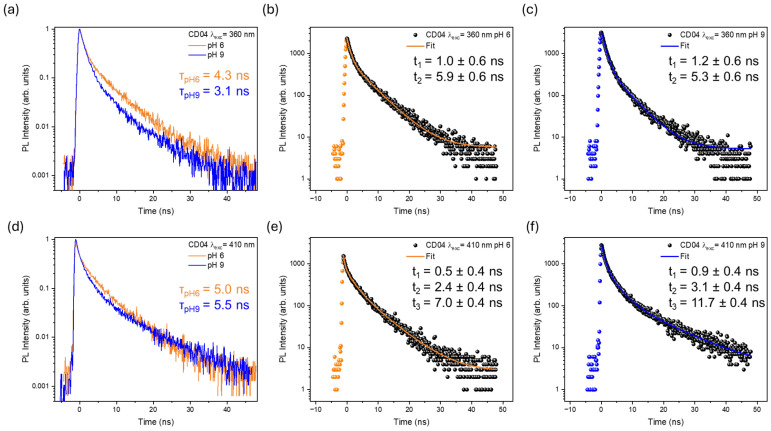
Decay time plots with relative calculated average lifetimes of CD04 excited at 360 nm (**a**) and 410 nm (**d**) at pH 6 (in orange) and pH 9 (in blue). The corresponding multiple-exponential fits are reported for each decay: at 360 nm and pH 6 (**b**), at 360 nm and pH 9 (**c**), at 410 nm and pH 6 (**e**), and at 410 nm and pH 9 (**f**). All decay times were extracted from the overall emission spectrum.

We successfully measured the decay plots of CD01, CD02, CD04, and CD05. TR-PL measurements for CD03 could not be recorded because its excitation channels were unavailable for our instrumentation. CD01 and CD02 were excited at both 360 and 500 nm, while CD04 and CD05 were excited at both 360 and 410 nm (Figure S7 and Figure 7). All estimated decay times were obtained by calculating a weighted average lifetime [91], as extracted from a non-single exponential decay fit assuming two decay components (three for CD04 at 410 nm). The time resolution was evaluated based on the signal’s 10–90% rise time, which is 0.6 ns for excitation at 360 nm and 0.4 ns for excitation at 410 and 500 nm, with all decays investigated over a 50 ns time window except for CD05 at 360 nm, with a resolution of 1.1 ns over 100 ns time window (Figures S8 and S9).

For CD01 excited at 360 nm, the average lifetime of the overall emission increases from 3.2 ns to 5.3 ns (Figure S7a,d). A similar trend is observed with excitation at 500 nm, where the longer lifetimes are correlated with the relative increase in PL intensity. In this case, the relative extension of the lifetime is larger (from 0.8 to 5.7 ns) than at 360 nm due to the larger increase in the differential PL intensity (Figure 5). Indeed, at 500 nm, we observed almost total suppression of the emission at pH 5.

Conversely, CD02 shows different behaviors depending on the excitation wavelength, reflecting the different nature of its two emission bands at 475 nm and 545 nm (Figure S7b,e). The calculated lifetime of the emissions excited at 360 nm is 3.1 ns at both pH 6 and pH 9, with no variation within the estimated uncertainty. When it is excited at 500 nm, a general increase in lifetime is observed (from 0.8 to 1.6 ns). The longer lifetime recorded at pH 9 is paired with the larger increase in emission intensity compared to pH 6, indicating that the 500 nm excitation wavelength is more efficient than the 360 nm one in sensing the environment changes.

CD05 (Figure S7c,f) exhibits an opposite trend compared to CD01 and CD02, with the lifetime decreasing as the pH increases for both 360 nm and 410 nm excitations. The estimated lifetime (Figures S8 and S9) drops from 8.5 ns to 5.9 ns when excited at 360 nm and from 7.2 ns to 4.7 ns under 410 nm excitation, despite the overall small increase in the emission signal. As already discussed, this sample presents larger nanoparticles, and the emission bands are possibly related to different synthesis products and aggregation phenomena, leading to a plethora of pH-sensitive edge centers.

Among the samples, CD04 proved, once again, to be the most interesting sample, exhibiting opposite trends at different excitation wavelengths. When excited at 360 nm (Figure 7a), the average emission lifetime decreases from 4.3 ns to 3.1 ns due to a relative change in the weight of each component. Indeed, the analysis of the individual time decays (Figure 7b,c) revealed that each decay time did not change within the error margin. Thus, the observed variation is related to their relative contribution to the average decay. A different phenomenon could be observed when the sample was excited at 410 nm (Figure 7d), where the decays required a three-exponential fit. The average value slightly increased from 5.0 ns at pH 6 to 5.5 ns at pH 9. Whilst the fastest lifetime component remained constant within the estimated uncertainty, the other two components were shorter at pH 6 compared to pH 9, confirming the different nature of the emission bands involved (Figure 7f,g). Indeed, looking at the maps in the differential PL intensity (Figure 5), the emission band at 500 nm, when excited at 360 or 410 nm, displays an opposite trend (increasing and decreasing, respectively) compared to its ratiometric counterpart (420 and 630 nm, respectively). This suggests the possibility of designing a ratiometric pH sensor exploiting two different excitation wavelengths to further increase its pH sensitivity.

Finally, it is interesting to note that the optical properties of the carbon dots can be associated with variations in their zeta potential across different pH levels, providing insights into the surface charge behavior under varying conditions. Indeed, changes in surface charge with pH can influence the optical properties of the nanoparticles by altering their aggregation state, which, in turn, can affect the linearity of the fluorescence. The dependence of surface potential on pH and the corresponding isoelectric point (I.E.P.) of the different CD samples were determined through a zeta potential (ζ) titration, as shown in Figure S10 and Table S1. The different titration curves observed for the five CD samples are due to different $pK_{a}$s of the ionizable functional groups occurring at the CD surfaces. The type, strength, and surface density of these groups are likely dependent on the chemical nature of the reagents used in the CD synthesis and on the particular synthetic procedure.

For instance, CD03 has a zeta potential of about −30 mV in the pH range of 4.5–9.0, which would result in a high colloidal stability of CD dispersions in water. On the other hand, CD04 might cause aggregation, particularly at a slightly acidic pH (I.E.P = 5.6). However, at higher pH values, CD04 would be colloidally stable, since it reaches a ζ = −25 mV at pH 9.6. Both the CD04 and CD05, the only samples to show ratiometric properties, showed large variations in the zeta potential across the investigated pH range (Figure S11), whilst CD01, CD02, and CD03 displayed an almost constant value of surface electric charge. In addition, our best candidate, CD04, was characterized by the largest variation in the zeta value, also suggesting that the aggregation of nanoparticles should be taken into account to explain the optical changes as a function of pH. Overall, the trend in zeta potential across different pH levels proved to be a good indicator of the surface characteristics of our nanoparticles, which can influence the linearity of PL intensity and consequently the suitability of the sensor.

## 4. Conclusions

The aim of this research was to explore the potential application of a series of CD samples as ratiometric fluorescent probes for pH sensing. All the investigated samples proved to be effective as traditional fluorescent sensors in both the UV and visible wavelength regions, displaying significant variations in fluorescence intensity when shifting from pH 6 to pH 9 in an aqueous-based Carmody buffer solution.

Notably, incorporating m-phenylenediamine in the synthesis enabled the transition from standard fluorescent sensors to ratiometric ones. This resulted in CDs with distinct emission bands whose fluorescence intensities changed oppositely as a function of pH. This precursor is known for its high reactivity, facilitating the formation of CDs through cross-linking, carbonization, or polymerization reactions. Additionally, the high nitrogen content in the nanoparticle structure contributes to the protonation and deprotonation of surface-related groups, which can be linked to the ratiometric behavior of this type of CD.

In particular, CD04 exhibited pH sensitivity at various excitation wavelengths in the UV (265, 305, and 345 nm) and visible regions (475 nm). The emission bands in the near UV and visible regions are especially relevant for practical applications, as these wavelengths are commonly available in commercial devices. Indeed, by exciting at 345 nm and 475 nm, we were able to linearly track the variation in pH by measuring the intensity ratio of emissions from two different centers in the visible range ($\lambda_{\mathrm{exc}}$ = 345 nm: $I_{435\;\mathrm{nm}}$/$I_{510\;\mathrm{nm}}$ and $\lambda_{\mathrm{exc}}$ = 475 nm: $I_{525\;\mathrm{nm}}$ /$I_{630\;\mathrm{nm}}$ ). Furthermore, the green emission, which is common to both ratiometric evaluations and undergoes an opposite variation with respect to its ratiometric counterpart, suggests the possibility of designing a novel sensor that exploits dual excitations to enhance sensitivity.

Overall, these findings highlight the potential of using carbon dots as ratiometric pH sensors in aquatic environments. Future research should focus on optimizing various factors, such as concentration, to enhance their practical applicability in environmental monitoring and sensor technology. Additionally, testing these ratiometric sensors in real seawater environments will be crucial to evaluate their performance and reliability under real-world conditions.

## Data Availability

All relevant data can be found in the article and its Supplementary Information files.

## Supplementary Materials

The following supporting information can be downloaded at: https://www.mdpi.com/article/10.3390/nano14171434/s1, Figure S1: PL spectra of the five CDs; Figure S2: PLE spectra of the main emission channels of the five CDs; Figure S3: Excitation and emission maps of the five CDs at low pH (left) and high pH (right); Figure S4. PL spectra of CD01 (a), CD02 (b), CD03 (c), and CD05 (d) excited at different excitation wavelengths at pH 5 (in red), pH 6 (in orange), and pH 9 (in blue); Figure S5: Excitation and emission maps of CD04 at pH 7 (a) and pH 8 (b); Figure S6: Gaussian deconvolution of the emission spectra of CD04 excited at 475 nm at pH 6 (a) and pH 9 (b); Figure S7: Decay time plots showing the relative calculated average lifetime of CD01 (a,d), CD02 (b,e), and CD05 (c,f) excited at 360 nm, 410 nm, and 500 nm; Figure S8: Decay time plots with relative multiple-exponential fit for CD01 (a,d), CD02 (b,e), and CD05 (c,f) excited at 360 nm. The plots are shown for low pH conditions (top) and high pH conditions (bottom); Figure S9: Decay time plots with relative multiple-exponential fit for CD01 (a,d), CD02 (b,e), and CD05 (c,f) excited at 410 nm (in blue) and 500 nm (in green). The plots are shown for low pH conditions (top) and high pH conditions (bottom); Figure S10: Experimental zeta potential (ζ) of CD samples versus pH; Figure S11: Experimental zeta potential (ζ) of CD samples as a function of a pH range from 6 to 9; Table S1: Isoelectric point of CD samples obtained through zeta potential/pH titrations.
