# Supplementary material for: Ratiometric Fluorescent pH Sensing with Carbon Dots: Fluorescence Mapping across pH Levels for Potential Underwater Applications
Source: Nanomaterials (Basel). 2024 Sep 2;14(17):1434. doi: 10.3390/nano14171434 (PMC11397204; doi:10.3390/nano14171434)
Supplement: Supplementary file 1 [file nanomaterials-14-01434-s001.zip › nanomaterials-3163677-supplementary.pdf]

Supporting Information

Section 1: Optical Properties

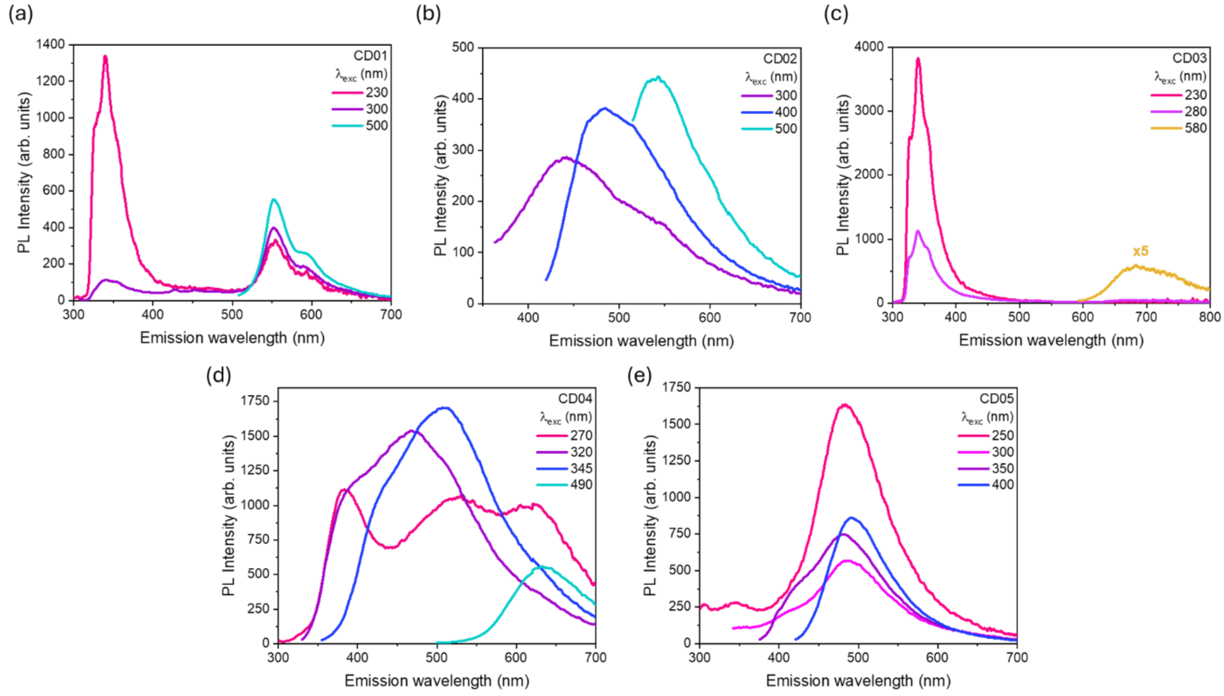

Figure S1. PL spectra of CD01 (a), CD02 (b), CD03 (c), CD04 (d) and CD05 (e) excited at their main excitation wavelengths.

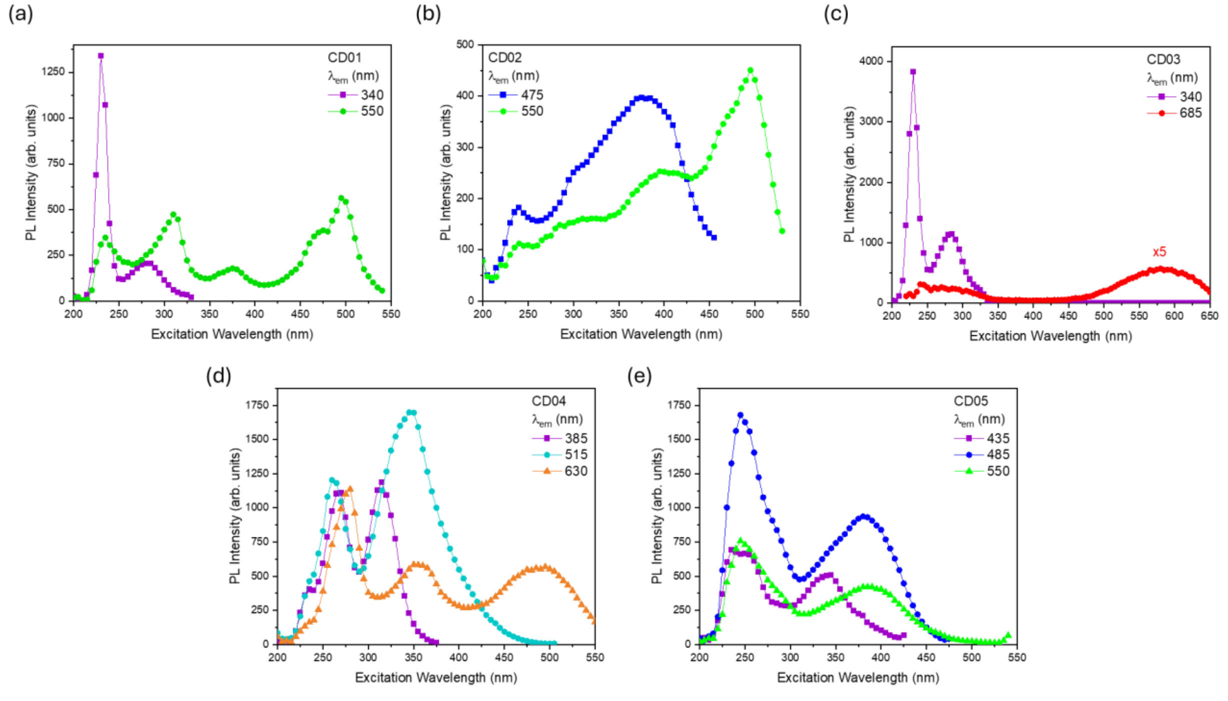

Figure S2. PLE spectra of the main emission channels of CD01 (a), CD02 (b), CD03 (c), CD04 (d) and CD05 (e).

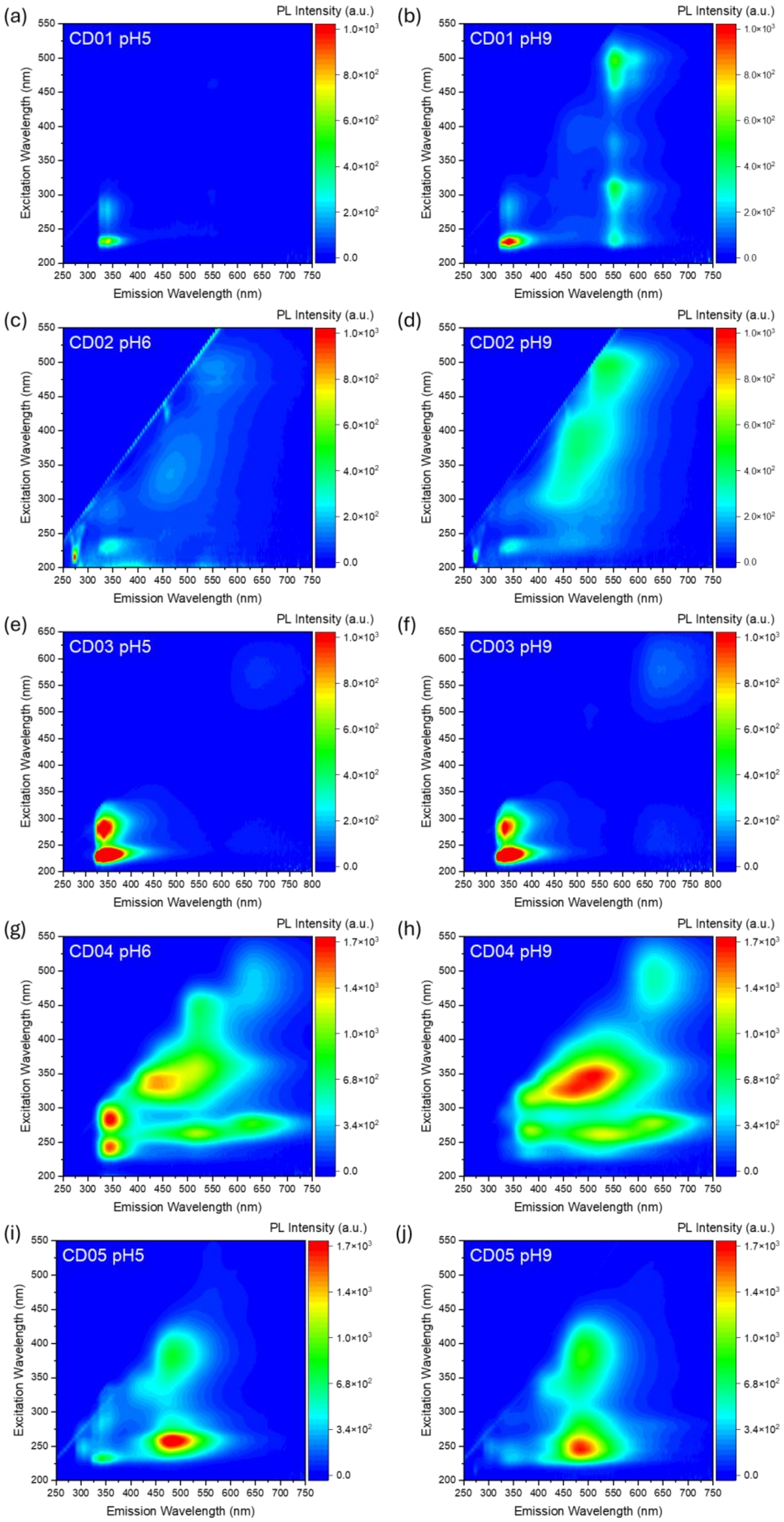

(a)
CD01 pH5
(b)
CD01 pH9
(c)
CD02 pH6
(d)
CD02 pH9
(e)
CD03 pH5
(f)
CD03 pH9
(g)
CD04 pH6
(h)
CD04 pH9
(i)
CD05 pH5
(j)
CD05 pH9
Excitation Wavelength (nm)
Emission Wavelength (nm)
PL Intensity (a.u.)

Figure S3. Excitation and emission maps of CD01 (a,b), CD02 (c,d), CD03 (e,f), CD04 (g,h), and CD05 (i,j) at low pH (left) and high pH (right).

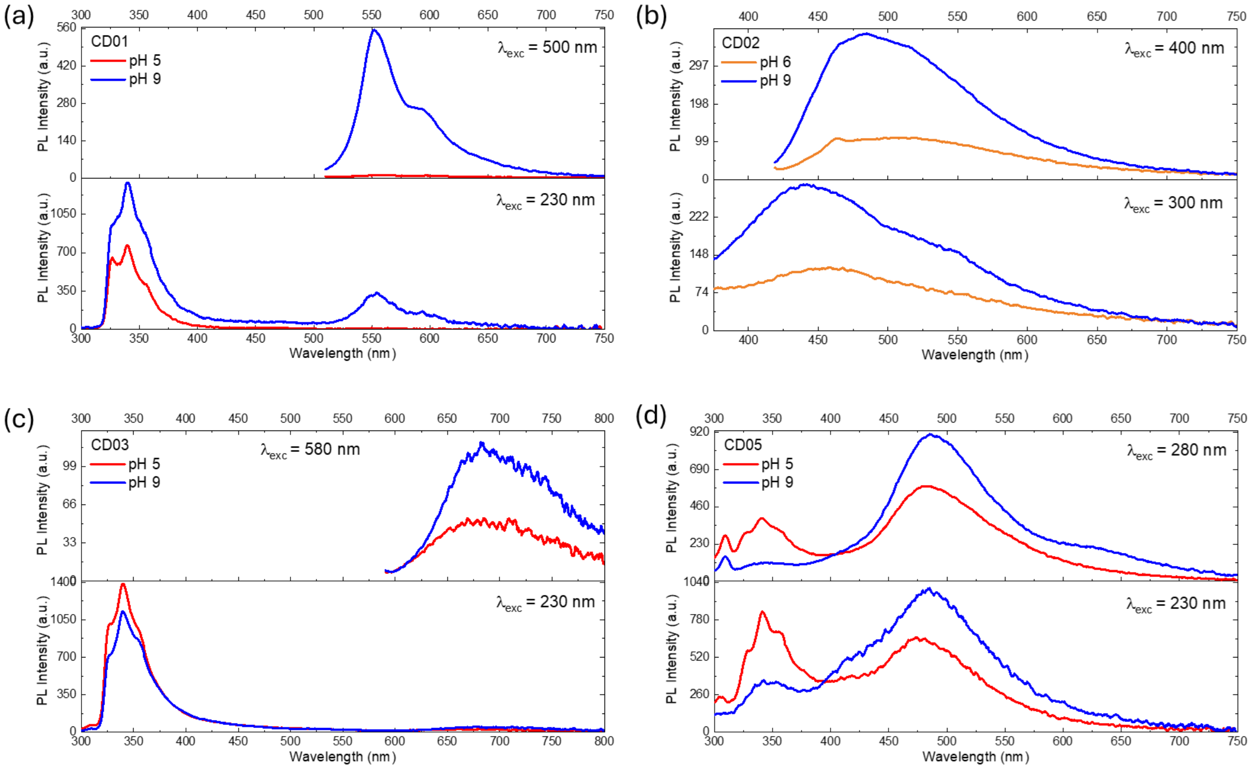

Figure S4. PL spectra of CD01 (a), CD02 (b), CD03 (c), and CD05 (d) excited at different excitation wavelengths at pH 5 (in red), pH 6 (in orange), and pH 9 (in blue).

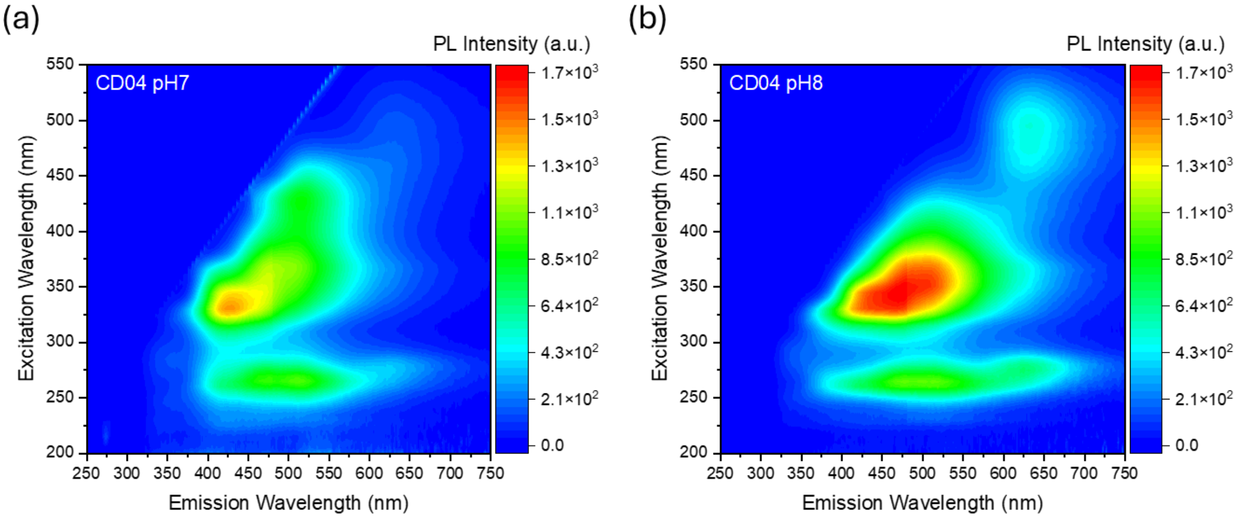

Figure S5. Excitation and emission maps of CD04 at pH 7 (a) and pH 8 (b).

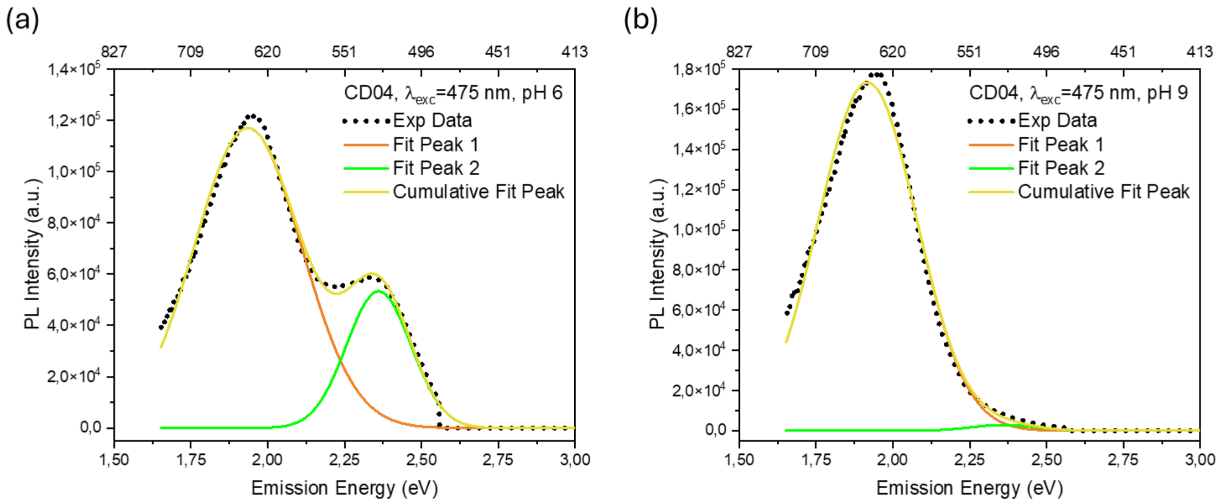

Figure S6. Gaussian deconvolution of the emission spectra of CD04 excited at 475 nm at pH 6 (a) and pH 9 (b). The R-square of the fit was 0.995 and 0.997 respectively. The spectra were corrected for the PL intensity using the Jacobian factor.

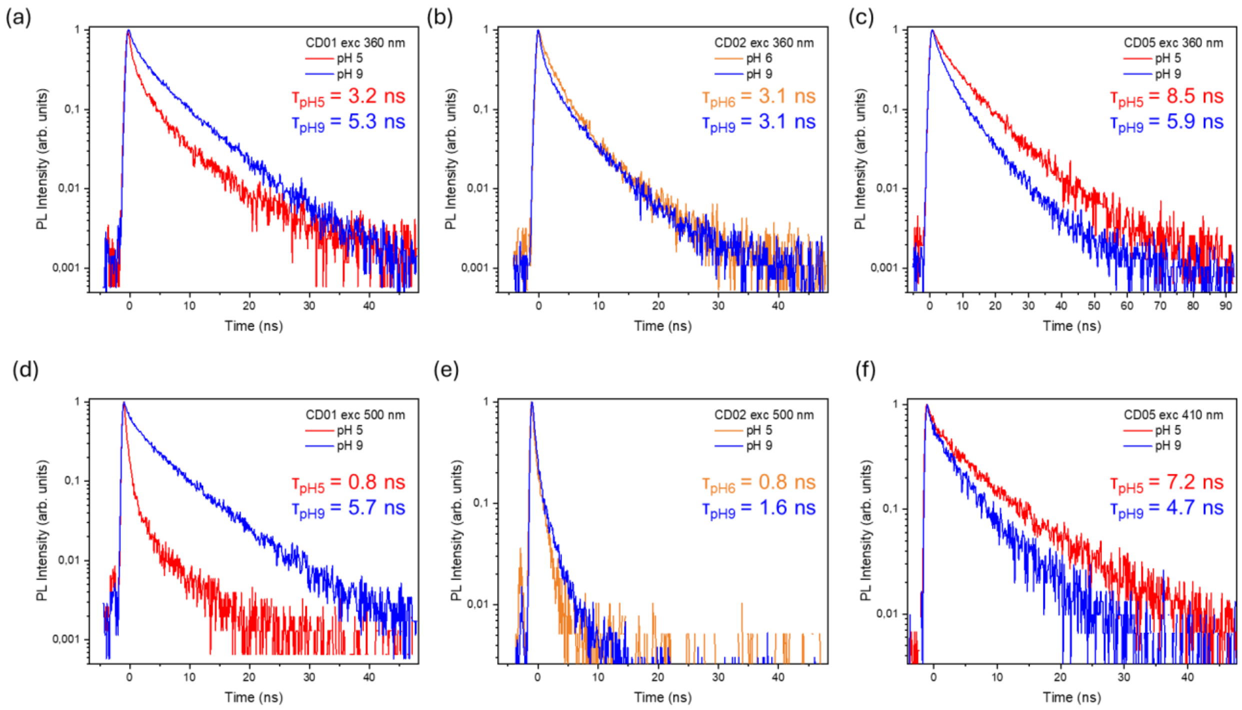

Figure S7. Decay time plots showing the relative calculated average lifetime of CD01 (a,d), CD02 (b,e), and CD05 (c,f) excited at 360 nm, 410 nm, and 500 nm. The data are presented for pH 5 (red), pH 6 (orange), and pH 9 (blue). The decay time was recorded on the overall emission spectrum.

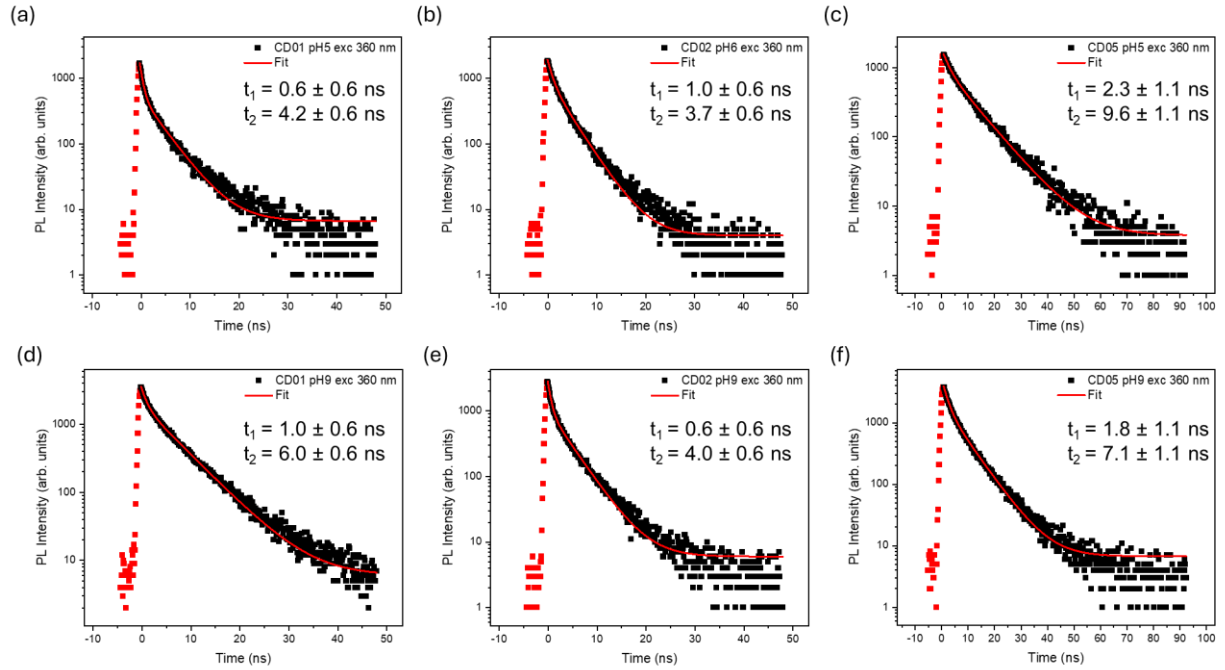

Figure S8. Decay time plots with relative multiple-exponential fit for CD01 (a,d), CD02 (b,e), and CD05 (c,f) excited at 360 nm. The plots are shown for low pH conditions (top) and high pH conditions (bottom). The decay times were recorded across the entire emission spectrum.

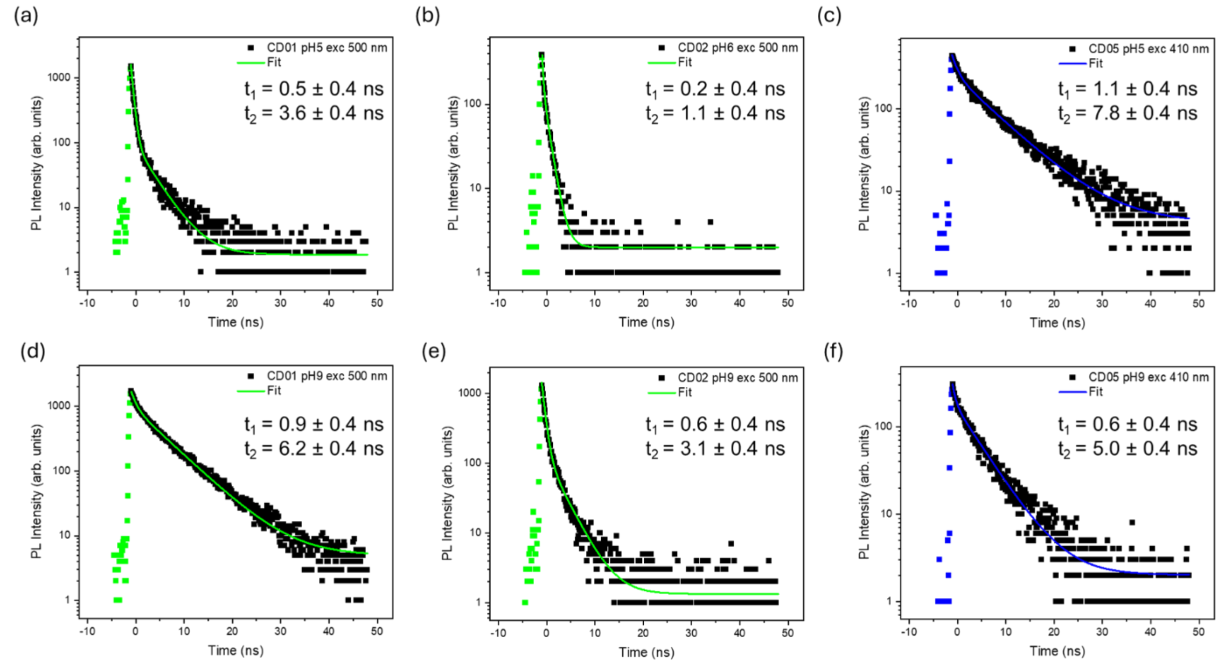

Figure S9. Decay time plots with relative multiple-exponential fit for CD01 (a,d), CD02 (b,e), and CD05 (c,f) excited at 410 nm (in blue) and 500 nm (in green). The plots are shown for low pH conditions (top) and high pH conditions (bottom). The decay times were recorded across the entire emission spectrum.

Section 2: Zeta potential

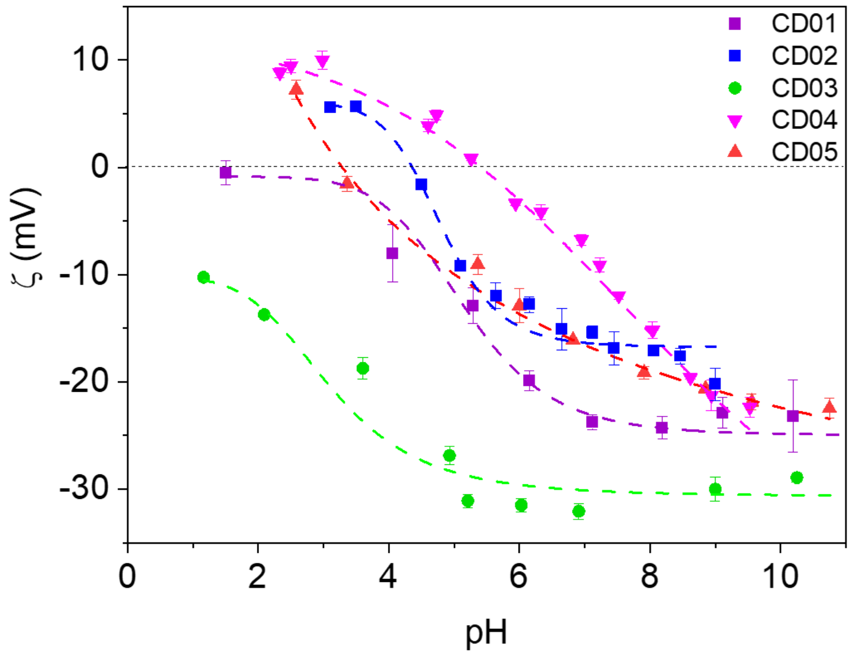

Figure S10. Experimental zeta potential (ζ) of CD samples versus pH.

Table S1. Isoelectric point of CD samples obtained through zeta potential/pH titrations.

**Sample**	**Isoelectric point**
CD01	1.5
CD02	4.3
CD03	<1
CD04	5.6
CD05	3.3

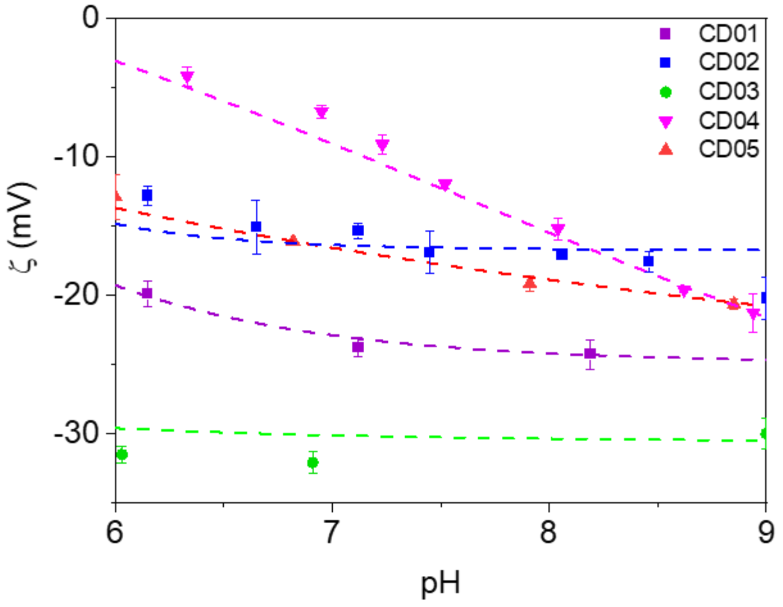

Figure S11. Experimental zeta potential (ζ) of CD samples as a function of a pH range from 6 to 9.
